# Gastric schwannoma with post-surgical gastroparesis: a case report and literature review

**DOI:** 10.3389/fonc.2024.1496074

**Published:** 2025-01-13

**Authors:** Ganggang Miao, De Zhang, Jiajing Li, Yanxiang Deng, Xingwei Gu, Tingting Feng

**Affiliations:** ^1^ Department of General Surgery, The People’s Hospital of Danyang, Affiliated Danyang Hospital of Nantong University, Danyang, Jiangsu, China; ^2^ Department of General Surgery, The Affiliated Nanjing Hospital of Nanjing Medical University, Nanjing, Jiangsu, China; ^3^ School of Clinical Medicine, Wannan Medical College, Wuhu, Anhui, China; ^4^ Central Laboratory, The Fourth Affiliated Hospital of Nanjing Medical University, Nanjing, Jiangsu, China

**Keywords:** gastric schwannoma, surgical therapy, post-surgical gastroparesis, management of gastroparesis, therapeutic methods

## Abstract

Gastric schwannoma is a relatively rare submucosal mesenchymal tumor with low probability of metastasis and arises from Schwann cells of the gastrointestinal nervous plexus. Surgical therapy is the main treatment of gastric schwannoma with symptoms or malignant tendency. Gastroparesis is a potential complication following gastrointestinal surgery, which is a clinical syndrome caused by gastric emptying disorder and characterized by nausea, vomiting, and bloating, resulting in insufficient nutrient intake. Generally, post-surgical etiology is the main potential etiology of gastroparesis, while the most common underlying etiology is diabetes mellitus. So far, reports of gastroparesis arising from resection of gastric schwannoma are rare. We present an 80-year-old woman who was diagnosed with gastrointestinal stromal tumor (GIST) primarily and has undergone laparoscopic wedge-shaped gastrectomy. The pathological and immunohistochemical examination ultimately established the diagnosis of gastric schwannoma. The patient experienced belching, nausea, vomiting, and bloating 1 week after the surgery and confirmed as gastroparesis through gastrointestinal series and gastroscopic examination. A series of treatments were performed, including correcting fluid-electrolyte disorders and vitamin deficiencies, and nutritional support and pharmacological treatments. The patient ultimately recovered well, and the relevant literatures were reviewed to identify and handle similar cases hereafter.

## 1 Case report

**Figure 1:**
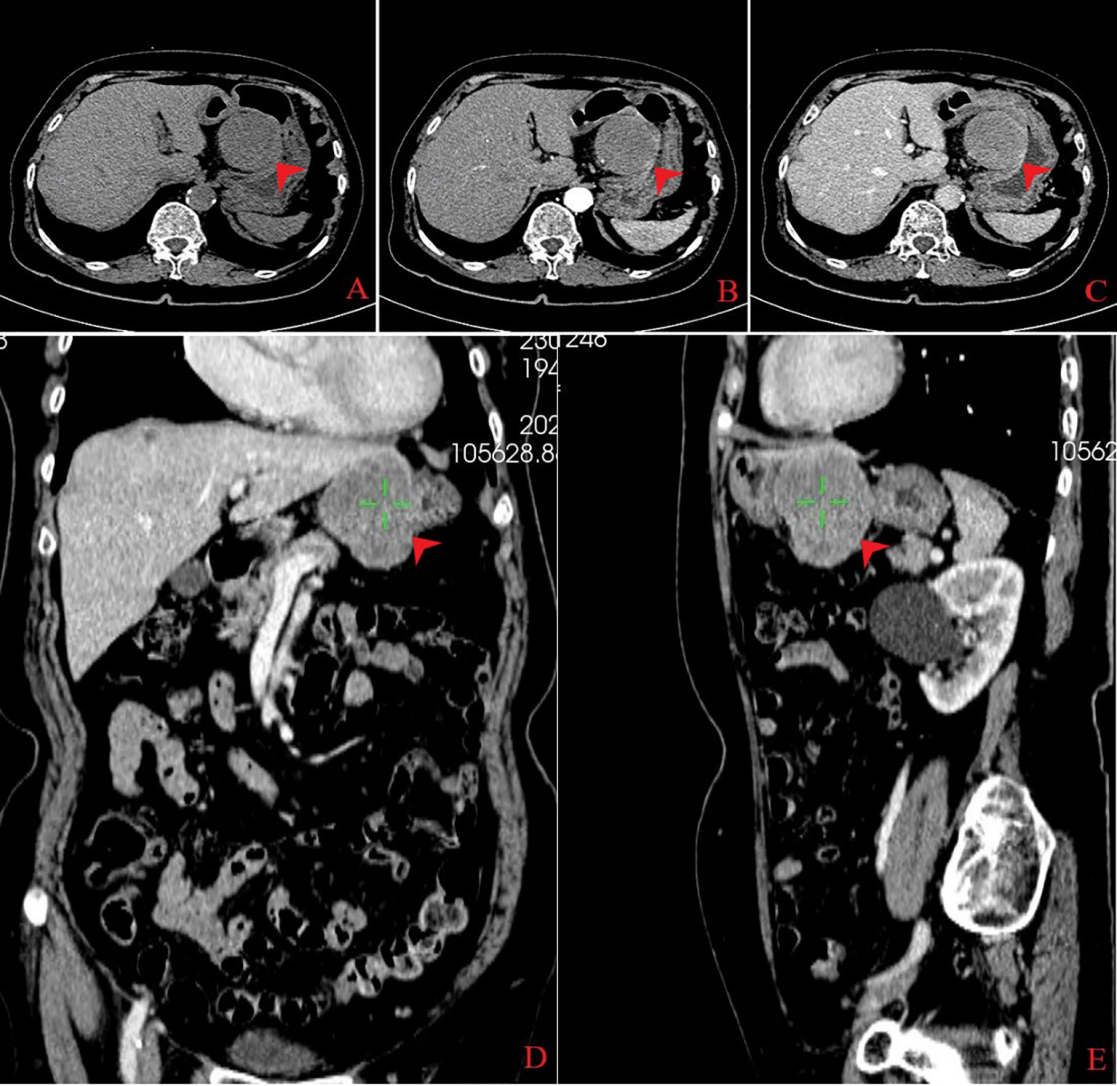
Computed tomography findings. An exophytic mass measuring 5.5 cm × 5.1 cm  × 5.0 cm was found in the lesser gastric curvature from three dimensions (transverse, coronal, and sagittal sections), which exhibited low density on plain computed tomography **(A)**. The mass was homogeneous enhanced in the arterial phase **(B)** and markedly, homogeneously enhanced in the venous phase **(C–E)**.

**Figure 2:**
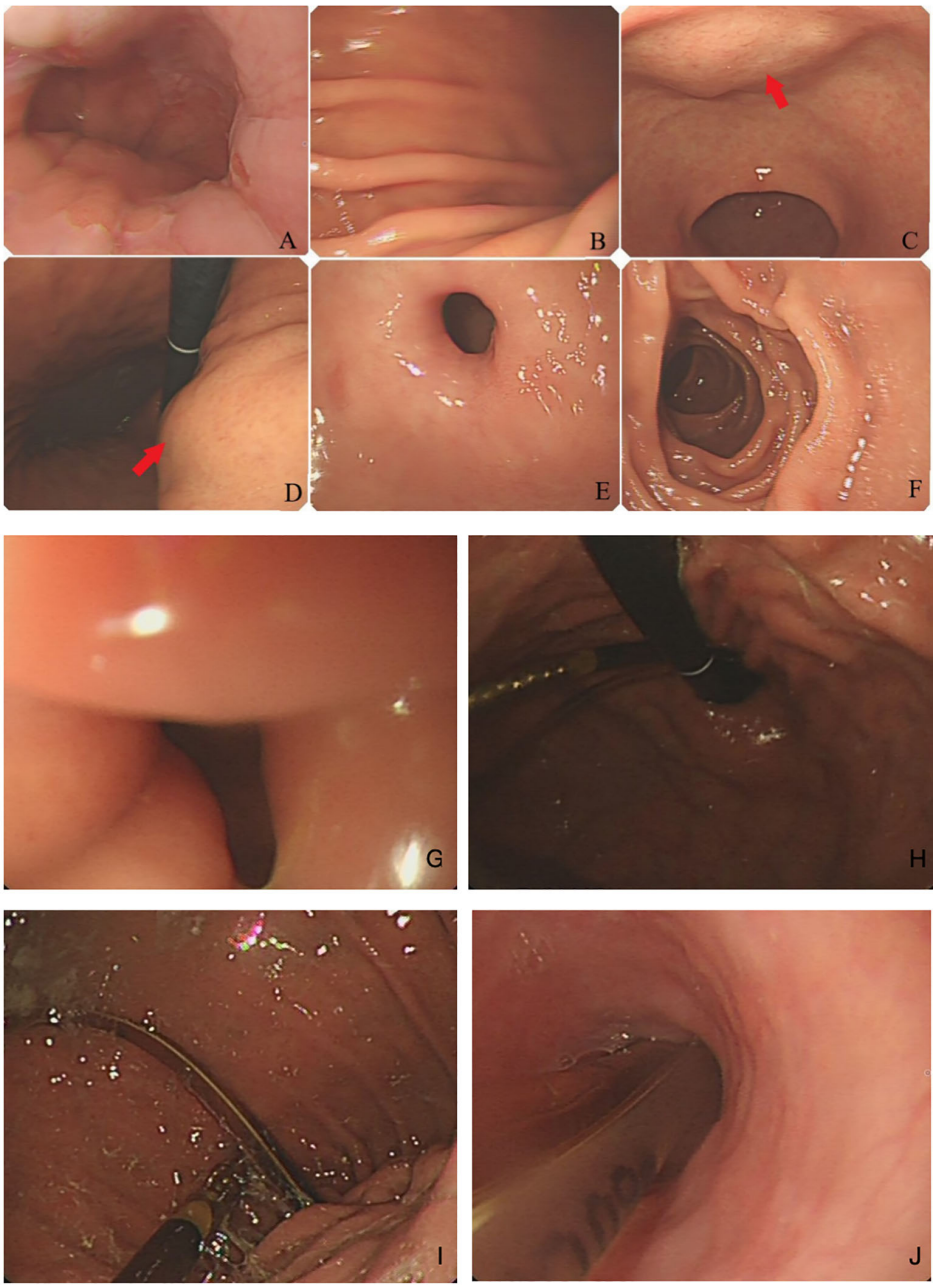
Gastric endoscopy: **(A)** esophagogastric junction; **(B)** greater gastric curvature; **(C, D)** lesser gastric curvature, a submucosal tumor without mucosal erosion; **(E)** pylorus; and **(F)** descendant duodenum. **(G)** pyloric edema with stenosis, **(H, I)** gastric dilatation, and **(J)** placement marking of the jejunal feeding tube.

An 80-year-old woman was admitted to the hospital due to the discomfort in the upper abdomen for approximately 1 month; physical examination and the laboratory tests revealed no abnormities, and some of the preoperative hematonic and biochemical results are summarized in **Table 1**. The plain and contrast-enhanced computed tomography (CT) showed an exophytic mass at the lesser curvature of the gastric antrum, which was enhanced and measured approximately 5.5 cm × 5.1 cm  × 5.0 cm. The boundary was blurred, while the mucosal tissue seemed to be uninvolved (**Figure 1**). Gastroscopy showed a submucosal mass at gastric antrum with good mobility, which caused the disappearance of the mucosal folds without mucosal erosion (**Figure 2**). Based on these findings, the primary diagnosis was GIST, which is the most common tumor that originated from the gastrointestinal mesenchymal tissue.

**Table 1:** The basic information and hematinic/biochemical results on admission.

Age	80 years old
Gender	Female
BMI	24.84
ASA class	Class II
White cell count	4.54 × 10^9^
Neutrophils	2.89 × 10^9^
Lymphocytes	1.26 × 10^9^
Monocytes	0.32 × 10^9^
Hemoglobin	128 g/L
Platelet	232 × 10^9^
Potassium	4.02 mmol/L
Sodium	143.0 mmol/L
Chlorine	105.0 mmol/L
Urea nitrogen	6.49 mmol/L
Creatinine	60.7 µmol/L
Uric acid	229.8 µmol/L
Conjugated bilirubin	3.6 µmol/L
Alkaline phosphatase	81.6 U/L
Gamma-GGT	13.2 U/L
Alanine transaminase	13.1 U/L
Aspartate transaminase	13.8 U/L
Lactate dehydrogenase	181.5 U/L
T lymphocytes	894 M/L
B lymphocytes	283 M/L

**Figure 3:**
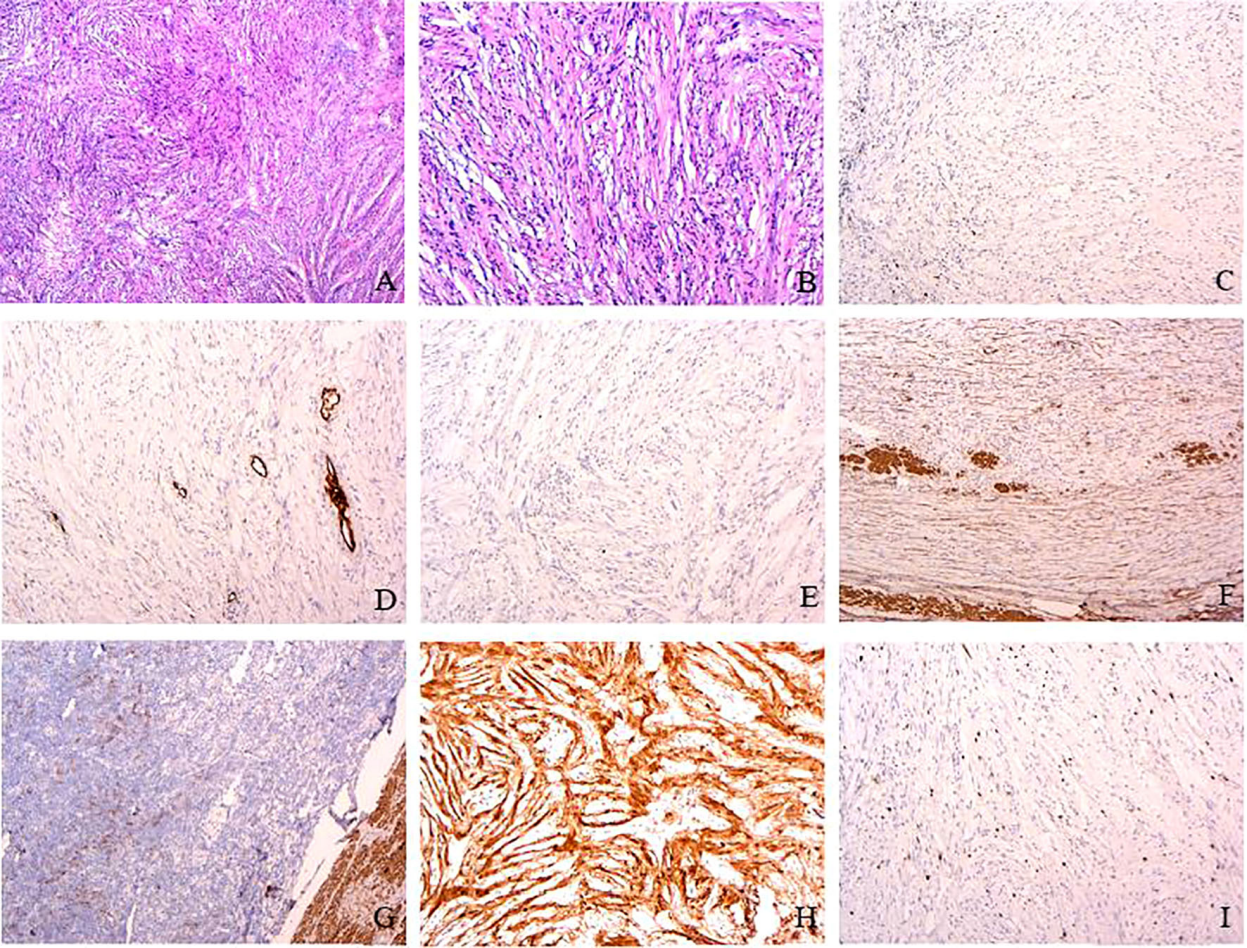
Pathological findings. Hematoxylin–eosin staining revealed that the mass was made of spindle cells with nuclei arranged in a palisading (HE×4, **A**) and the spindle cell proliferation with a lack of atypia, necrosis, and mitosis (0–2/50 field mitotic count) (HE×10, **B**). Immunohistochemistry revealed positive staining of S-100 (**H**) and negative staining of CD117 **(C)**, CD34 **(D)**, Dog-1 **(E)**, SMA **(F)**, desmin **(G)**, and Ki-67 (<2%) **(I)**. S-100 protein is a marker widely found in neural crest-derived cells, including Schwann cells, pigment cells, and adipocytes. In this case, the positive S-100 staining of the tumor further supports the diagnosis of gastric schwannoma. CD 34 is present at the level of the vascular endothelium. SMA and desmin present in the smooth muscle layer of intratumoral vessels (magnification, ×10).

The patient underwent laparoscopic wedge-shaped gastrectomy with transtracheal general anesthesia. Pathological examination revealed that the mass was made of spindle cells, with nuclei arranged in a palisading, and a lack of atypia, mitosis, and necrosis. Immunohistochemistry revealed positive staining of S-100 and negative staining of CD117, CD34, Dog-1, desmin, and Ki-67(<2%) (**Figure 3**); S-100 protein is a common immunohistochemical marker, which is expressed in many cells derived from neural crest, including Schwann cells, pigment cells, and adipocytes. Immunohistochemical positivity of S-100 protein is an important supporting evidence in the diagnosis of gastric schwannoma. However, the accurate diagnosis needs to be combined with the histopathological characteristics of the tumor and the expression of other immunohistochemical markers (such as CD117, CD34, and Dog-1) to exclude other types of tumors such as gastrointestinal stromal tumors and leiomyomas, which further confirmed a gastric schwannoma (1).

**Figure 4:**
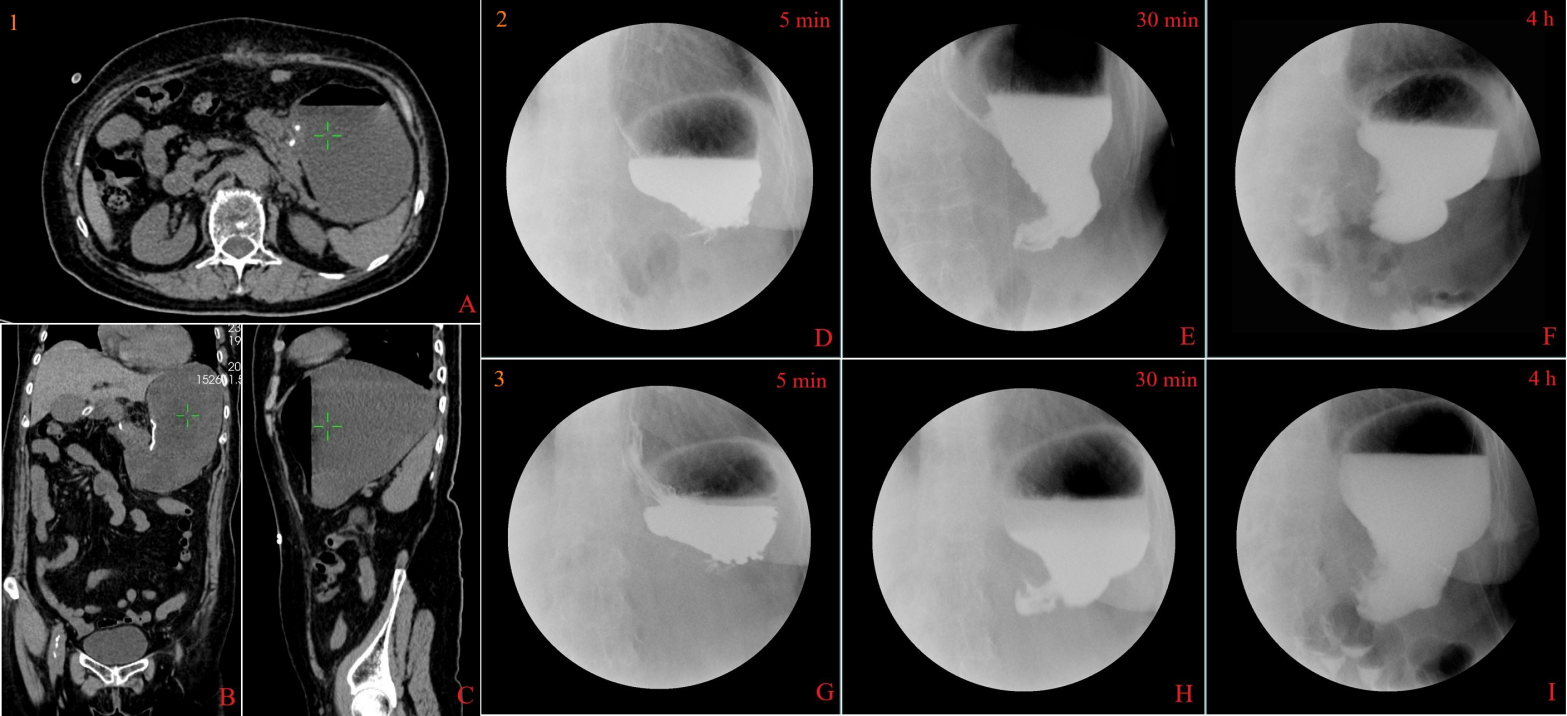
Computed tomography and X-ray findings. Abdominal computed tomography scan showed significant dilation of the gastric cavity, accumulation of digestive fluid, and no indication of pyloric or duodenal stenosis (the beginning of gastroparesis, **1A–C**). Upper gastrointestinal series indicates disappearance of gastric peristalsis, with the contrast media accumulated and delayed emptying for more than 4 h in different periods(the beginning of gastroparesis, **2D–F**; 2 months of gastroparesis, **3G–I**).

The patient experienced belching, nausea, vomiting, and bloating 1 week after the surgery. Abdominal CT revealed significant expansion of the gastric cavity with accumulation of digestive fluid, and upper gastrointestinal series indicates disappearance of gastric peristalsis, with the contrast media accumulated and delayed emptying for more than 4 h (**Figure 4**). Meanwhile, gastroscopy examination indicates dilation of the gastric cavity, lack of motility, and swelling of the pylorus with open–close normally (**Figures 2G-J**).

The nasojejunal tube and nasogastric tube were quickly placed with DSA guidance when the postsurgical gastroparesis was confirmed. However, the refractory gastroparesis lasted for more than 3 months. During this period, appropriate liquid and electrolytes were provided to maintain the fluid–electrolyte balance; parenteral nutrition and enteral nutrition were added to correct nutritional deficiency. Meanwhile, some pharmacological agents, including metoclopramide, mosapride, mecobalamin, and vitamins, were supplemented; some indicators are summarized in **Table 2**. So far, the patient has recovered well without any digestive symptoms during the follow-up period.

**Table 2:** The dynamic changes in some hematinic/biochemical indicators.

Date	WBC (×10^9^)	Hb (g/L)	ALB (g/L)	TP (g/L)	A/G ratio	Drainage volume of gastric juice (mL/day)
05.06.2023	4.54	128	40.7	67.7	1.5	_
08.06.2023	10.14	126	36.7	63	1.4	10
12.06.2023	5.32	116	32.4	59.4	1.2	300
17.06.2023	7.04	123	35.3	65.2	1.2	400
23.06.2023	6.46	117	33.4	62.1	1.2	500
28.06.2023	6.49	123	35.2	65.5	1.2	460
05.07.2023	5.13	110	34.3	64.7	1.1	400
12.07.2023	5.32	107	33.5	61.7	1.1	400
24.07.2023	4.47	118	40.0	75.0	1.1	450
15.08.2023	4.09	110	37.7	66.2	1.3	700

## 2 Discussion

### 2.1 Gastric schwannoma

Gastric schwannoma is a neurogenic submucosal gastric tumor and originates from the gastrointestinal neural plexus, which is relatively rare and easily confused with GIST, leiomyoma, and other neurogenic tumors (1, 2). The primary diagnosis of the case that we reported was GIST, which was ultimately confirmed based on histological and immunohistochemical examination. Previous studies have shown that gastric schwannoma accounts for approximately 2.6% of gastric mesenchymal neoplasms (3) and 0.2% of all gastric tumors (4).

Gastric schwannomas are a kind of slow‐growing benign tumor, which can be malignant in 13.8% of cases as well (5, 6). Tao et al. reviewed 28 patients from a research institute in China who had no recurrence or metastasis within a median follow-up time of 50 months, and the long-term postsurgical results were good (7). A retrospective analysis of Li et al. showed that 32 surgical cases of gastric schwannoma had no recurrence or metastasis during the follow-up period (4–96 months; mean, 35 months; median, 23 months) (8).

Radiological and endoscopic techniques play a significant role in describing the localization, size, and morphological characteristics of the tumor; however, they cannot differentiate accurately among GIST, myogenic, and neurogenic tumors (2, 9). Endoscopic ultrasound with biopsy is an advanced method for confirming the diagnosis by pathology, despite associated with the risk of perforation and tumor metastasis (3, 10). The diagnosis of schwannoma is mainly based on histological and immunohistochemical examination; histological examination shows that the cells have spindle-shaped nuclei and fascicular arrangements with immunohistochemical positivity of S-100 protein, which is a marker of neoplastic Schwann cells (1, 2, 11). Generally, surgical therapy is the core in most cases without any other adjuvant therapy for the benign property. Surgical resection, including laparoscopic or laparotomic partial gastric resection, may be considered safe and effective. Laparoscopic resection may be the preferred option for most small- and medium-sized tumors (7). With the development of endoscopic technology, endoscopic therapy has gradually become a novel option, which includes endoscopic submucosal dissection, endoscopic full thickness resection, and ligation-assisted endoscopic removal (12, 13). However, it is limited by the tumor size (>3.0 cm) and location within the muscularis propria due to the increased risk of perforation and hemorrhage (3, 11, 14).

Considering the size, location, and surrounding structure of the tumor, a laparoscopic wedge resection was performed in our case. Although the surgical process was very successful, the patient experienced refractory post-surgical gastroparesis.

### 2.2 Post-surgical gastroparesis

The etiology of gastroparesis varies. Diabetes and post-surgery are common causes, which account for 29% and 13%, and up to 36% of the cases are of idiopathic etiology (15); potential causes include myopathic diseases, neurological diseases, or direct tumor infiltration (16, 17).

Postsurgical gastroparesis is one of the complications of gastrointestinal surgery (18); the mechanism remains unclear nowadays. Gastroparesis and impaired gastric accommodation may result from neuromuscular dysfunction, such as smooth muscle dysfunction, enteric/autonomic nervous system abnormalities, gastrointestinal surgery-related decreased motilin hormone, vagotomy, and tumor infiltration; in addition, immune-mediated destruction of the interstitial cells of Cajal is one of the proposed mechanism (19, 20).

Although the symptoms or nutrient requirement may be gradually alleviated in the patients with mild gastroparesis, those with refractory gastroparesis typically require one or more following measures: intravenous hydration, correction of vitamin deficiencies and metabolic disorders (ketoacidosis and hypoglycemia), reduction in nasogastric pressure, and/or nutritional support, pharmacological therapy, and interventional treatments (21, 22).

### 2.3 Correcting fluid–electrolyte disorders and vitamin deficiencies

Refractory vomiting and/or severe inadequate intake will lead to fluid–electrolyte imbalance (potassium, zinc, iron, and calcium), deficiencies in vitamins (A, B6, B12, C, and K), and metabolic disorders (23, 24). Evidence from the National Institutes of Health Gastroparesis Federation showed that up to 64% of gastroparesis patients have insufficient dietary calories, which is <60% of their estimated total energy requirements (17, 24). Therefore, it is necessary to maintain fluid–electrolyte balance and correct vitamin and mineral deficiencies through reasonable means, such as intravenous injection or nasogastric tube.

### 2.4 Nutrition support

The patients suffering from refractory gastroparesis are unable to consume sufficient calories through oral intake and may become malnourished eventually; the stepwise nutritional interventions are recommended (25, 26).

#### 2.4.1 Parenteral nutrition

Parenteral nutrition is used as a bridging therapy for the patients, which is suitable for patients with severe weight loss, contraindications to enteral nutrition, insufficient nutritional needs, and poor oral tolerance, as long-term parenteral nutrition can cause centerline infections, metabolic complications and thrombosis (25, 27).

#### 2.4.2 Enteral nutrition support and nasojejunal tube

Inadequate intake may be of a more prolonged and/or intermittent nature due to gastroparesis. Although dietary interventions and medications are the first line of treatment, enteral nutrition support may be necessary to maintain adequate nutritional status (28).

It is common practice to place a nasojejunal feeding tube to deliver enteral nutrition, which has been shown to be safe and effective (25, 29). A few days of nasojejunal feeding trials should precede the infusion rate with at least 60mL/h. It is important to allow for several days of habituation and gradually increase infusion rate from 10 mL/h to 60 mL/h, as some patients have coexistent intestinal dysmotility or may not tolerate the rate of calorie infusion. Moreover, if long-term treatment of enteral nutrition support is required, percutaneous jejunal feeding has been proven to be safe and effective (30, 31).

### 2.5 Pharmacological therapy

Gastric emptying rate is an assessed indicator for patients with gastroparesis, and most of the patients experienced upper gastrointestinal symptoms, such as belching, nausea, vomiting, and bloating, although the correlation between delayed gastric emptying and symptomatic severity is generally poor (32). Once the diagnosis of gastroparesis is confirmed, treatment should focus on the predominant symptoms; the first-line pharmacological therapy has always been the prokinetic and antiemetics agents (33, 34).

#### 2.5.1 Prokinetics agents

Prokinetic agents are the mainstay of pharmacological treatment; clinical trials have shown efficacy in enhancing gastric emptying and reducing symptoms of gastroparesis. which include dopamine receptor antagonists (metoclopramide and domperidone), 5-HT4 agonists (cisapride and mosapride), and macrolides (erythromycin, azithromycin, and clarithromycin) (17, 32, 33, 35–38).

Metoclopramide, a peripheral cholinergic and antidopaminergic agent, is the only US FDA-approved medication for the treatment of gastroparesis. However, the long-term use is restricted by a decline in efficacy and the side effects on the central nervous system, including tardive dyskinesia (30, 33, 39).

Domperidone is a peripheral dopamine D2 receptor antagonist that is available for gastroparesis, and the effect is comparable to metoclopramide. However, domperidone was associated with the corrected QT interval prolongation (17, 40–42).

Cisapride is a 5-HT4 agonist and accelerates gastric emptying of solids and improves dyspeptic symptoms in gastroparesis; it prolongs the QT interval and causes torsade de pointes and ventricular tachycardia (43).

Macrolide antibiotics have agonistic effects on motilin receptor and accelerate gastric emptying; motilin is a peptide hormone that promotes activation of smooth muscles in L-type calcium channels (44). The prokinetic effects of erythromycin have been positive even when compared to metoclopramide (45). Long-term antibiotic use may be associated with complications, including antibiotic resistance and potential infections (46).

#### 2.5.2 Antiemetic agents

Antiemetic agents have been used to relieve the symptoms of gastroparesis, which were used to reduce vomiting symptoms through blocking dopamine type-2 (D2) receptor (dopamine antagonists), 5-HT3 receptor (serotonin antagonists), histamine type-1 (H1) receptor (antihistamines), and muscarinic receptor (anticholinergics) or neurokinin type-1 receptors (neurokinin antagonists) (47).

The benzamide derivatives as previously mentioned are prokinetics, which also exert an antiemetic effect by inhibiting dopamine receptors in the brainstem (47, 48). 5HT-3 receptor antagonists such as granisetron and ondansetron are effective in controlling chemotherapy-induced nausea and vomiting (49).

Ondansetron is a 5-HT3 receptor antagonist that reduces nausea from stomach distension without affecting gastric compliance, volume, or accommodation (50). Ondansetron causes QTc prolongation and, in rare cases, can lead to torsades de pointes, a life-threatening cardiac arrhythmia (51).

Respiratory depression and sedation are common side effects of prochlorperazine, promethazine (H1-receptor antagonists) (52, while sedation, dry mouth, and blurry vision, and central cholinergic syndrome are common side effects of scopolamine(M-receptor antagonists) (53).

#### 2.5.3 Novel and experimental medications

Botulinum toxin is a potent neuromuscular conduction inhibitor, which was used for ameliorating achalasia symptoms by reducing esophageal sphincter pressure (54). Experiments have confirmed that injecting botulinum toxin into the pylorus can ameliorate symptoms of delayed gastric emptying and inhibit smooth muscle contraction by reducing the response to acetylcholine (55).

In addition, some experimental medications were developed for treatment, including tradipitant (NK1 antagonist), felcisetrag (5-HT4 agonist), trazpiroben (dopamine D_2_/D_3_ receptor antagonist) and relamorelin (ghrelin agonist) (56–59).

### 2.6 Interventional treatments

#### 2.6.1 Surgical treatment

Generally, gastrectomy should generally be discouraged, which was considered as the “end of the road situations” where no other intervention has proved helpful. Surgical gastrectomy can be considered for selected patients with postsurgical gastroparesis. Surgical intervention measures are usually reserved for patients with gastroparesis after vagotomy (60, 61).

##### 2.6.1.1 Gastrectomy

Subtotal or total gastrectomy with Roux-en-Y gastric bypass can be proposed as the ultimate surgical option, but a cautious approach is warranted before surgical therapies in diabetic or idiopathic gastroparesis (62, 63).

##### 2.6.1.2 Surgical pyloroplasty

This technique is mainly performed by using laparoscopic approach, and the most famous technique is Heineke Mikulicz, which is characterized by a longitudinal incision of the pyloric ring and transverse suture. Almost 90% of patients reached an improvement or the normalization of the gastric emptying. In addition, robotic pyloroplasty has been recently proposed as a safe and effective approach (63, 64).

##### 2.6.1.3 G-POEM

Inspired by the treatment of achalasia by POEM, G-POEM was introduced into clinical practice. In refractory gastroparesis, G-POEM is superior to a sham procedure for improving both symptoms and gastric emptying 6 months after the procedure. These results are not entirely conclusive in patients with idiopathic and postsurgical etiologies (65). G‐POEM is a safe technique with encouraging clinical efficacy as treatment for patients with refractory gastroparesis. Targeting the pylorus seems to be effective at accelerating gastric emptying and thus leads to symptomatic improvement of nearly 60%–80% of patients at mid-term follow-up. Validation of the technique in further randomized trials is highly expected to confirm the initial results (66).Regardless of the cause, up to one-third of patients do not improve in postoperative interventions. G-POEM is a minimally invasive therapy with long-term effectiveness and safety in treating postsurgical gastroparesis (67).

#### 2.6.2 Gastric electric stimulation

Gastric electric stimulation (GES) was considered as an available therapy for treating refractory gastroparesis. As early as 1997, the research result of GES conducted on animals and selected patients was published (68, 69). Several studies have demonstrated a clinical response to GES in patients with postsurgical gastroparesis. GES has led to great symptom relief in severe refractory postsurgical gastroparesis. A multicenter study involving 38 patients found that a significant reduction in weekly vomiting and nausea was observed at 4 weeks, and a 90% reduction in nausea and vomiting frequency was observed at 11 months after receiving percutaneous and late permanent GES treatment (70). A pilot study by Oubre et al. showed that GES led to weekly vomiting improvements and a reduction in total symptoms (71). A study by McCallum et al. further demonstrated improved symptoms, quality of life, nutritional status, and hospitalization requirements (72). GES may be considered among the therapies available for treating patients with refractory symptoms of gastroparesis and combined with pyloromyotomy for refractory gastroparesis (73).

##### 2.6.2.1 Placement of an electrical stimulator

A small stimulator characterized by high frequency (12 cycles/min) and low stimulation energy can be placed on the greater curvature of the stomach, 10 cm far from pylorus, with a laparoscopic or laparotomic approach (74). High frequency/low energy gastric electrical stimulation provided by implantable devices has been used in patients with refractory symptoms, with benefits in terms of symptomatology, gastric emptying, nutritional supplementation, and quality of life (75). Mechanisms of action remain poorly understood; although the symptomatic improvement is not related to gastric emptying acceleration (74), the changes in sympathetic vagal autonomic activity and thalamic pathway effects have been observed through electromyographic regulation (76).

#### 2.6.3 Acupuncture

Acupuncture has an application history of thousands of years in China and is becoming more popular in western countries due to its remarkable efficacy and few side effects (77). Acupuncture is a non-pharmacological intervention, which has the advantage of not being affected by herb–drug interaction and has been proven to be effective on postsurgical gastroparesis syndrome in both basic research and clinical practice (78). The mechanism may be related to the activation of sympathetic efferent nerve fibers or vagal nerve fibers, regulating the secretion of gastrointestinal hormones and reducing damage to interstitial cells of Cajal (ICCs) (79).

Postoperative gastroparesis is a complex and heterogeneous disease, and its prognosis varies with the patient’s basic health status, operation mode, lesion location, and timeliness and effectiveness of postoperative management. Literature shows that most patients with postoperative gastroparesis gradually relieved their symptoms within 6 months after operation, but some patients (especially severe or intractable cases) may need long-term treatment support.

The recovery of gastroparesis is directly related to the regenerative ability of gastric neuromuscular function. Gastric plexus injury or vagus nerve dysfunction is one of the potential causes, so early accurate diagnosis and individualized intervention are very important. Mild patients can achieve symptom control through nutritional support and drug therapy, while for refractory cases, emerging technologies such as gastric electrical stimulation (GES) and endoscopic pyloric myotomy (G-POEM) have shown remarkable therapeutic effects. According to a multicenter study, GES plays a significant role in relieving symptoms, improving nutritional status, and improving quality of life, with a success rate of 60%–80%. As a minimally invasive surgical technique, G-POEM can improve the gastric emptying rate by relaxing pyloric muscles, which provides an effective choice for patients with intractable gastroparesis.

In addition, the long-term management of postoperative gastroparesis should also include psychological support and comprehensive evaluation of quality of life. The education of patients and their families also plays an important role in improving treatment compliance. Future research should focus on the key predictors of gastroparesis recovery and how to optimize intervention measures to improve the treatment effect.

## 3 Mechanisms linking gastric schwannoma surgery and gastroparesis

In this case, the development of refractory post-surgical gastroparesis following gastric schwannoma resection can be attributed to several possible mechanisms related to tumor characteristics and the surgical approach. Gastric schwannomas, while rare, are neurogenic tumors originating from the gastrointestinal neural plexus. The tumor’s location near the lesser curvature of the gastric antrum and its significant size (5.5 cm × 5.1 cm × 5.0 cm) likely played a pivotal role in postoperative outcomes. Proximity to critical structures such as the vagus nerve and the pylorus increases the risk of disruption to neural and smooth muscle function, which are essential for coordinated gastric motility.

The surgical procedure—a laparoscopic wedge gastrectomy—may have exacerbated these effects. Although minimally invasive techniques aim to preserve surrounding structures, resection in areas with dense neural innervation, such as the gastric antrum, might impair the interstitial cells of Cajal or result in localized inflammation and scarring, further compromising gastric emptying. Additionally, the necessity to manipulate the pyloric region during tumor resection could have led to transient or permanent functional changes. Future studies should investigate whether tumor characteristics (e.g., size and exact location) and specific surgical techniques are significant predictors of post-surgical gastroparesis. This understanding could guide preoperative planning and risk mitigation strategies.

A thorough literature search revealed that reports of gastroparesis directly associated with gastric schwannoma management are exceedingly rare. Surgical management—the primary treatment modality—can lead to complications such as gastroparesis, although this is rarely documented. This suggests that while gastroparesis is not a common sequela of gastric schwannoma surgery, cases like ours highlight the need for awareness of its possibility, particularly in elderly patients or those with larger tumors.

The rarity of such reports underscores the significance of this case. It emphasizes the need for careful perioperative assessment and documentation of functional outcomes following surgical interventions for gastric schwannomas. Including this case in the existing literature provides valuable insights into the potential risks and management strategies for such complications.

## 4 Clinical experience and enlightenment

### 4.1 Preoperative evaluation of the potential impact of tumor location on postoperative complications

When gastric schwannoma is located near the vagus nerve and pylorus, it may significantly affect gastric motility. The accurate location of tumor and its relationship with peripheral nerves and tissues should be comprehensively evaluated by imaging and endoscopy before operation to provide basis for surgical planning.

### 4.2 Early identification and management of postoperative gastroparesis

The clinical manifestations of gastroparesis include belching, nausea, vomiting, and abdominal distension, which should be closely monitored in the early postoperative period. If necessary, confirm the diagnosis by gastrointestinal radiography and gastroscopy, and take timely intervention measures, including correcting electrolyte disorder, starting nutritional support, and using prokinetic drugs to prevent symptoms from getting worse or complications.

### 4.3 The key role of multidisciplinary teamwork

The diagnosis, surgical treatment, and postoperative management of gastric schwannoma need the cooperation of surgeons, pathologists, and gastrointestinal motility experts. The combination of accurate preoperative diagnosis, intraoperative technical optimization, and postoperative individualized treatment is helpful to improve the prognosis of patients.

### 4.4 Pay attention to long-term follow-up to evaluate the risk of postoperative recovery and recurrence

Gastric schwannomas are mostly benign, but there is still a rare possibility of malignancy. Postoperative follow-up cannot only monitor the recovery of gastric motility but also find potential recurrence or complications in time and provide continuous support for patients.

### 4.5 The importance of literature review

Although postoperative gastroparesis is rare in cases of gastric neurilemmoma, in-depth understanding of previous related cases is helpful to identify susceptible patients and optimize management strategies. The literature review of this case provides valuable reference for the diagnosis and treatment of similar cases in the future.

## 5 Conclusion

Generally, gastric schwannoma and gastroparesis are two diseases with minimal correlation. Gastric schwannoma is a rare and mostly benign tumor, but with malignant tendency as well. Surgical therapy is the main treatment for gastric schwannoma with symptoms or malignant tendency, including laparoscopic, laparotomic, or endoscopic approaches, although there is still controversy over the different surgical approaches. Although relevant studies have been conducted in many medical centers, there are few reports of postoperative gastroparesis caused by the management of gastric schwannoma, which may be related to the location and size of the tumor, proximity to the vagus nerve, and smooth muscle and pyloric dysfunction. Through the literature reviewed in this case, a profound understanding of gastric schwannoma and postoperative gastroparesis has been achieved.

## Data Availability

The original contributions presented in the study are included in the article/supplementary material. Further inquiries can be directed to the corresponding author.
